# PROTOCOL: School‐based language, math, and reading interventions for executive functions in children and adolescents: A systematic review

**DOI:** 10.1002/cl2.1262

**Published:** 2022-07-12

**Authors:** Jens Dietrichson, Morten Kjær Thomsen, Julie Kaas Seerup, Martin Williams Strandby, Bjørn Christian Arleth Viinholt, Elizabeth Bengtsen

**Affiliations:** ^1^ VIVE—The Danish Center for Social Science Research Copenhagen Denmark; ^2^ Department of Sociology University of Oxford Oxford UK; ^3^ VIVE—The Danish Center for Social Science Research Copenhagen Denmark

## Abstract

This is the protocol for a Campbell systematic review. Our primary objective for this systematic review is to examine if preschool and school‐based interventions aimed at improving language, literacy, and/or mathematical skills increase children's and adolescents' executive functions. As a secondary objective, we will examine how the effects of language, literacy, and mathematics interventions on executive functions are moderated by the subject of the intervention, child age or grade, the type of EF measured, and the at‐risk status of participants. We will also explore how the effects are moderated by other study characteristics, and estimate the effects of the included interventions on language, literacy, and mathematical skills.

## 1 BACKGROUND

### 1.1 Description of the condition

Recent reviews have found a substantial association between children's executive functions (EF) and their academic achievements (Blair, 2007; Follmer, 2018; Jacob, 2015; Peng, 2016; Peng, 2018; Peng, 2020). However, we are yet to understand the underlying reasons for this association. Is it because training EF skills also improves academic skills, or because training academic skills improves EF skills, or because other variables are influencing both academic and EF skills?

EF are a set of cognitive skills and processes used when directing behaviour towards the attainment of a certain goal (Miyake, 2000; Zelazo, 2015). Whereas EF is a term referring to multiple advanced cognitive skills and processes, core EFs generally include inhibitory control, working memory, and cognitive flexibility (e.g., Diamond, 2013). Inhibitory control is the control of one's attention, behaviour, thoughts, and emotions (Diamond, 2013). Working memory is the processes involved in the control, regulation, and active maintenance of task‐relevant information in the service of complex cognition (Castles, 2018). Cognitive flexibility is the ability to change perspective and adjust in the face of changed circumstances (Diamond, 2013; McClelland, 2012). Cognitive flexibility builds on inhibitory control and working memory, and develops later in life than the other two skills (Diamond, 2013). Thus, there is overlap between EF skills as well as between measures used in their assessment (Miyake, 2000). Furthermore, closely related concepts like self‐regulation (i.e., the ability to control and direct one's attention, thoughts, emotions, and actions) involves the application of all three core EF skills (Hernández, 2018; McClelland, 2012).

Numerous studies have found associations between EFs and a variety of desirable outcomes throughout life (see Diamond, 2013; for an overview). In addition to academic achievement, such outcomes include career success (Prince, 2007), marriage satisfaction (Eakin et al., 2004), and physical and mental health (Moffitt, 2011). Therefore, researchers and practitioners across fields have taken an interest in the nature of these associations, and ultimately, how EFs might be trained and what the effect of such training might be. A large number of research interventions as well as commercial products build on the premise that when measures of EF predict desirable outcomes, then training EF skills should improve such outcomes (Simons, 2016). However, this assumption of a causal effect from training EF skills has little empirical support: while training EF skills typically improves performance on the trained tasks, there is little compelling evidence that such training substantially improves performance on more distantly related tasks like academic achievement or general cognitive performance (for reviews see e.g., Cortese, 2015; Jacob, 2015; Katz, 2018; Melby‐Lervåg, 2013, 2016; Rapport, 2013; Redick, 2015; Sala, 2017; Schwaighofer, 2015; Shipstead, 2012; Simons, 2016).

In conclusion, we know that EF and academic achievements are associated, but so far there is little robust evidence that strengthening EF will improve academic skills. Hence, new approaches to examining the EF/academic achievements association are necessary. Another hypothesis is that training academic skills improves EF. That is, the association is explained by a causal effect of academic skills on EF skills. Theoretically, such effects may be expected for at least three reasons: First, neurocognitive skills like EF develop when they are used (Zelazo, 2015), and, for example, learning to read, speak, and understand a language, and performing maths operations involve the use of EF skills (e.g., Castles, 2018; Clements, 2016; Peng, 2020). Second, training academic skills may create new cognitive routines that are useful also for solving EF tasks (Gathercole, 2019). Third, learning academic skills increase domain‐specific knowledge, which in turn may improve performance on EF tasks (Oberauer, 2018).

In this systematic review, we will examine the effects of preschool and school‐based interventions aimed at enhancing language, literacy, and mathematical skills on the development of the child/adolescent's EF. That is, does training either language, literacy, or math skills improve EF? A positive answer to this question would imply that training academic skills affect EF skills. A negative answer would, together with the numerous reviews indicating that the effects of training EF skills on academic achievement are small, make it more likely that a third set of skills is behind the association between EF skills and academic achievement. Thus, we believe that the results of this review will improve the understanding of the association between EF skills and academic achievement.

### 1.2 Description of the intervention

Interventions of interest to this review must be aimed, at least in part, at enhancing language, literacy or maths skills of children and adolescents attending pre, primary, secondary, or high‐school. Furthermore, interventions must be applied, at least in part, in a preschool or school setting and must be administered by teachers, teaching assistants, preschool teachers, or the like. For example, solely increasing the amount of homework or the amount of time parents are encouraged to train their children's skills at home will not constitute an eligible intervention. Interventions might vary in duration and methodology and do not have to be prevalidated in terms of having an effect on language, literacy, or maths skills.

While we will restrict the review in terms of the content of interventions (i.e., to language, literacy, or maths), the instructional methods used in the interventions will not be restricted. Examples of instructional methods are: tutoring or small‐group interventions by adults, peer‐assisted instruction by same‐age or older peers, whole‐class interventions, interventions that increase progress monitoring by using curriculum‐based measurements, or computer‐assisted interventions using software programs or apps. Interventions that change the content rather than format of instruction, for example by emphasising phonological awareness in early literacy training, will also be included as long as the aim is to improve language, literacy, or maths skills. Interventions must constitute a condition different from ‘treatment as usual’ (TAU), and, to avoid confounding components, interventions with the specific purpose of training EF skills will not be eligible for this review.

Interventions must be evaluated by a measure of EF. As numerous others have noted before us, researchers differ substantially in the specific dimensions they include and identify when conducting research on EF (e.g., Garon, 2008; Jacob, 2015; Jurado, 2007). In this review, we wish to include studies on EFs in the broad sense, meaning that measures of inhibitory control, working memory, and cognitive flexibility, as well as more composite skills, such as self‐regulation, will be included.

In summary, we expect to include a range of different preschool and school‐based interventions all aimed at increasing language, literacy, and/or maths skills and testing the effects of such interventions on validated measures of EF.

### 1.3 How the intervention might work

As training EF has limited or no effects on academic skills, how is the robust associations between measures of EF and academic achievement to be explained? One hypothesis is that academic skills and EF are both caused by one or more variables, which are associated with both academic skills (Brunner, 2021; Sirin, 2005) and EF (Jacob, 2015; Lawson, 2018). One example of such a variable may be parental socioeconomic status (SES). There is abundant evidence that growing up in high SES families, on average, provide environments more conducive to academic achievement than growing up in low SES families. These environmental advantages include richer language and literacy environments (Bus, 1995; Golinkoff, 2019; Hart, 2003), parents having higher expectations of their children's academic achievement (Bradley, 2002; Slates, 2012), and having better access to resources such as high‐quality early childhood education, health care, nutrition, and enriching spare‐time activities (e.g., Esping‐Andersen, 2012; Morgan, 2012). However, as there are also many low SES children and youth who thrive in school (Dietrichson, 2017), it may be the activities more often carried out in high SES families, rather than the SES per se, that influence child development (Lawson, 2018). Interestingly, activities that high SES parents typically do more of, such as child‐directed speech, reading together with their children, and helping children with their homework are typically aimed at improving language, literacy, and maths. That is, (pre)academic skills—not EF. Nevertheless, these activities might actually work as EF training sessions.

In line with this notion, a second hypothesis—the one we will examine in this review—is that training academic skills improves EF. One reason to expect such effects is that neurocognitive skills like EF develop when they are used; with repeated use, the neural circuits involved in the mental operation become more efficient (Zelazo, 2015). As learning to read, to speak, and understand a language, and performing maths operations involve the use of EF skills (e.g., Castles, 2018; Clements, 2016; Peng, 2020), academic interventions may improve EF skills. Furthermore, if more academic training involves more use of EF skills, then longer academic interventions may improve EF skills more than shorter interventions. However, the duration of the intervention may have countervailing effects because a long intervention for example increases stigma or make children demotivated (see e.g., Dietrichson, 2017; Dietrichson, 2021; Wanzek, 2006, 2013; for reviews finding negative or null associations between duration and effects on academic skills).

As demonstrated by the lack of effects from EF skills interventions on academic achievement, training one set of skills may not be enough to improve another set (James, 1890; Woodworth, 1901). In other words, improving skills on one task may not *transfer* to other tasks. Taatgen (2013) theorised that transfer between tasks will occur when training on one task develops a set of operators, which are also useful for a new task. Similarly, Gathercole (2019) argued that training one set of cognitive skills (in their case working memory) provides benefits to other skills when the training involves learning new cognitive routines that can be applied to novel or not‐yet‐learned tasks involving the other skill. ‘Near’ transfer to similar tasks are therefore more likely than ‘far’ transfer to dissimilar tasks (see Barnett 2002 for a typology of what constitutes near and far transfer). More training would only be an improvement up to the point where the new routine has been learnt, and would not transfer at all if the routine cannot be applied to the novel task.

Although the mechanisms that produce transfer between cognitive skills are not well‐understood (Katz, 2018; Simons, 2016), there are several candidates for how training language, literacy, and maths skills may transfer to EF skills. Self‐directed speech, the ‘outer speech’ used by young children to guide themselves while performing tasks and the ‘inner speech’ used by older children and adults, seems to be important for cognitive functions and the regulation of behaviour (e.g., Luria, 1959; Vygotsky, 1980). Better language skills could improve EF skills by enhancing self‐directed speech (Bishop, 2014; Weiland, 2014). For example, language skills may help children formulate more complex verbal rules that enable the remembering of task sequences and the activation of the relevant task set before operations (Cragg, 2010; Zelazo, 1998, 2015), or help children override overlearned responses in favour of a novel response, that is, to self‐regulate (Doebel, 2016; Luria, 1959). Doebel (2016) found that 3‐year‐olds who were exposed to contrasting negations (of the form ‘not X, Y’) scored higher on measures of EF skills than children who were either only exposed to contrasting stimuli, or read storybooks with an adult. Melby‐Lervåg (2010) found that second graders improved serial and free recall after training phoneme awareness and vocabulary, but not after training rhymes. They argued that improved phonological and semantic memory representations of the words, which rhyme training did not provide, may explain the pattern of results.

In sum, language, literacy, and math interventions may affect EF skills by creating new cognitive routines that transfer to EF tasks. If verbal processes play an important part in the development and exercise of self‐regulation and other EF skills, then interventions improving language skills may improve EF skills. As literacy training may improve phonological, vocabulary, and comprehension skills (Morrison, 2019; Stanovich, 1986), literacy interventions may therefore affect EF skills through similar channels. Learning mathematics involves training in logical and statistical reasoning, which have been shown to transfer to the solution of novel problems (e.g., Simons, 2016) and may in general involve the development of new cognitive routines that also can be applied to EF tasks (e.g., Clements, 2016; Demetriou, 2014).

Successful language, literacy, and maths interventions furthermore increase domain‐specific knowledge. Oberauer (2018) cited evidence that knowledge from past experience has substantial effects on the performance of working memory tests. Manifestations of this effect included that prior learning improve ‘chunking’ (i.e., combining items into larger sets), that known words are easier to remember than unknown, and that repetition improve performance. If academic interventions are successful, they improve learning and knowledge, which may therefore be a channel through which such interventions may improve working memory, and potentially, other EF skills. As for example cognitive flexibility builds on working memory skills (Diamond, 2013), improved domain‐specific knowledge may improve cognitive flexibility through the same channels.

The mechanisms explaining why academic interventions may affect EF skills do not rule out that training EF skills also improve academic skills. On the contrary, several authors have hypothesised a bidirectional relationship between academic skills and EF skills (e.g., Castles, 2018; Clements, 2016; Connor, 2016; Peng, 2020). A bidirectional, or reciprocal, relationship between academic skills and EF would seem to predict that training EF skills should also improve academic skills but, as mentioned, the causal evidence for this direction is not strong. However, the effects found in meta‐analyses of EF training programs (primarily working memory training) on academic achievement in children and youth are typically positive and small, not precisely estimated null effects (Cortese, 2015; Melby‐Lervåg, 2013; Melby‐Lervåg, 2016; Rapport, 2013; Sala, 2017; Schwaighofer, 2015). Thus, these reviews do not rule out small positive effects of training working memory on academic achievement.

Some theories emphasize the unidirectional relationship from academic skills to EF skills, or downplay the possibilities for transfer from training EF skills to academic skills (Demetriou, 2014; Gathercole, 2019). Gathercole (2019) argued that the new cognitive routines learnt through working memory training programs are unlikely to apply to language, literacy, and maths tasks. As academic skills rely on an extensive array of cognitive routines, they are unlikely ‘to be trained with anything other than real‐life experience’ (Gathercole, 2019, p. 38). Van Der Maas (2006) argued that changing one single variable (i.e., a single EF skill) in a complex system (such as academic skills) may be ineffectual. If transfer between skills depends on the content of learning, the similarity of the contexts in which that learning is applied, and the interaction between the content and context, then specific content would transfer less often (Simons, 2016). If language, literacy, and mathematical learning involves a more complex array of skills and the learning of more cognitive routines than EF training interventions typically have provided, then transfer from academic skills training to EF skills may be more likely than the other way around.

Longitudinal studies examining whether the relationship between EF and academic skills is uni‐ or bidirectional have for example found unidirectional associations from expressive vocabulary to EF skills (e.g., Jones, 2020), from EF skills to receptive vocabulary (Weiland, 2014), and from self‐regulation to reading achievement (Hernández, 2018). Bidirectional associations have been found between reading comprehension and self‐regulation (Connor, 2016) and maths achievement and self‐regulation (Hernández, 2018), while some studies have only found insignificant associations between the development of EF skills and language skills (Gooch, 2016). However, these longitudinal studies lack the exogenous variation in both EF and academic skills needed to identify a causal relation (Hernández, 2018; Jones, 2020). The interventions we will examine provide exogenous variation in one direction and will provide evidence of the effects of training language, literacy, and maths skills on EF skills.

As mentioned, the content and context of training may be important for transfer between skills (Barnett & Ceci, 2002). Clements (2016) argued that the relationship between mathematics and EF is stronger than between literacy and EF. In a similar vein, Hernández (2018) hypothesised that EF skills may be most useful when applied to novel situations, and maths, at least in primary and secondary school, may be less automatised than language and literacy processes. As language is not automatised when children are very young, and many literacy skills are not automatised until children are fluent readers, this hypothesis would also suggest age‐dependent effects.

The neural basis of EF skills is another reason to expect heterogeneity of effects across age. Although EF skills develop throughout adolescence (Diamond, 2013), the plasticity of the nervous system declines with age (e.g., Zelazo, 2015). Short‐term memory processes are essential for early skill acquisition but less important once the cognitive processes behind a skill have been automated (Van Der Maas, 2006). Early interventions may therefore have larger effects on EF skills. This discussion also suggests interaction effects between the type of intervention and age. For example, language interventions may have the largest effects on the EF skills of very young children and literacy interventions around the start of primary school when most children acquire basic decoding skills.

In their review, Peng (2020) found that reading and mathematics skills predict cognitive skills and vice versa, but that this bidirectional relationship was weaker for disadvantaged students. They hypothesised that the differences in learning experiences and opportunities between advantaged and disadvantaged children explain the weaker bidirectionality. That is, advantaged children or not‐at‐risk children, including high‐SES children, who start out with stronger cognitive and academic skills may be more likely to trigger and benefit from cognitive‐academic bidirectionality. A similar mechanism may be in play regarding the association between EF skills and language, literacy, and mathematics skills.

Study characteristics may also influence the effects (e.g., Cheung, 2016). A potential moderator is the type of control group. Using an active control group that performs similar activities as the treatment group in all aspects but the ‘working ingredient’ (i.e., a placebo condition) may be advantageous when the aim of a study is to pinpoint the mechanism through which an intervention affects a skill. Because a placebo condition may shut down other possible mechanisms, such as changed expectations and motivations, it increases the chances of isolating the hypothesised mechanism (Simons, 2016). However, in field experiments in preschools and schools, using a placebo condition is not necessarily an advantage. Education interventions, whether intentionally or not, may improve academic and EF skills precisely through the changes in motivation and expectations that using a placebo condition aims to preclude (Bøg, 2021; Diamond, 2014). Closing down these mechanisms by using a placebo condition may thus change what is being estimated. Moreover, TAU control groups may be more at‐risk of Hawthorne and John Henry‐effects: that the treatment and control groups behave differently because they know that they are participating in a study (Glennerster, 2013). While such effects are difficult to completely avoid in education interventions, placebo control groups also know that they are participating in study and, if the placebo treatment works well, believe that they participate on equal terms with the treatment group. Hawthorne and John Henry‐effects may therefore be mitigated.

As mentioned, the duration of the intervention is a potential effect size moderator. Further examples of study characteristics that may moderate effect sizes include the study design—whether it is a randomised controlled trial (RCT), quasi‐randomised controlled trial (QRCT), or a quasi‐experimental study (QES)—the type of measure (whether the children are measured directly, or assessed by someone who knows them well, like a teacher or a parent), and measurement timing. With the exception of measurement timing, the direction of the influence on effect sizes of these moderators is theoretically ambiguous.

In sum, language, literacy, and maths interventions may improve EF skills for at least three reasons: because learning to read, speak, and understand a language, and performing maths operations involve the use of EF skills; because training language, literacy, and maths create new cognitive routines that are useful also for solving EF tasks; and because the interventions improve domain‐specific knowledge. We will examine whether this theoretical promise is borne out empirically. As discussed in this section, there are reasons to expect effect sizes to be moderated by the subject of the intervention, the type of EF skill measured, child age or grade, the at‐risk status of participants, as well as study characteristics. We will examine these potential moderators in our investigation of heterogeneity (see the Subgroup analysis and investigation of heterogeneity section).

### 1.4 Why it is important to do this review

Education spending is a large proportion of total government spending in many countries around the globe (OECD, 2020). In the UK alone, education spending is the second‐largest element of public service spending, representing £90 billion in 2017‐18 (Belfield et al., 2018). EF skills are fundamental cognitive skills underlying all forms of goal‐directed behaviour (Miyake, 2000; Zelazo, 2015). With previous research identifying a strong association between EF and academic achievements (e.g., Jacob, 2015), educational policy makers have been right to take an interest in the potential training of these important cognitive skills within a school setting. However, researchers are yet to understand the nature of this association, and how it can be fully utilised for the benefit of the education system and ultimately its students.

The number of previous reviews of the effects of academic interventions on EF is small. Peng (2020) reviewed evidence of a bidirectional relationship between academic and more general cognitive skills (including EF). Their results suggest that reading and mathematics skills predict cognitive skills and vice versa, that this bidirectional relationship is weaker for disadvantaged students, and that direct academic instruction can improve cognitive skills. The review did not include a meta‐analysis. Their results provide motivation for conducting moderator analyses for advantaged and disadvantaged students.

In a narrative review of interventions in preschool and early primary school, Clements  (2016) found more evidence of an association between EFs and maths achievement than between EFs and literacy or language achievement. Furthermore, they cited studies showing reciprocal associations between early numeracy and EF, but not between early literacy and EF. These findings motivate us to examine whether the content of the interventions moderate effect sizes. Clements (2016) also cited studies finding unplanned effects on the EF of preschool children using the Building Blocks curriculum, which emphasizes mathematics (but not EF).

Less closely related, Ritchie (2018) presented meta‐analytic evidence that education can improve cognitive skills. Their meta‐analysis found that an additional year of schooling increases IQ with 1–5 points. Ritchie and Tucker‐Drob (2018) did not examine language, literacy or maths programs, or EF measures, and did not include preschool children. Stockard (2018) reviewed Direct Instruction interventions for school‐age children and found positive effects on IQ and cognitive skills measures. These measures were however not further defined in the review and it is unclear if any studies used similar measures as will be included in our review. Reviews of targeted and universal preschool programs have found effects on, typically broad, cognitive skills measures but not conducted analyses of EF skills (e.g., Dietrichson, 2020; Duncan, 2013; van Huizen, 2018).

We are not aware of a previous meta‐analysis examining the effects of academic interventions on EF skills. By examining the potential effect of preschool and school‐based language, literacy, and mathematics interventions on EF in children and adolescents, this review will contribute with important knowledge of the often highlighted relationship between EF and academic achievement. Therefore, this review contributes with (1) a reversed perspective on the association between EF and academic achievement from most earlier reviews; (2) a thorough and comprehensive risk of bias analyses of included studies; (3) an examination of intervention studies only, estimating the effect of training specific academic skills on the development of EF; (4) inclusion of several types of EF as well as both language, literacy, and maths interventions; and (5) a meta‐analysis on the above mentioned hypothesised association between academic interventions and EF.

## 2 OBJECTIVES

Our main research question for this systematic review is: *Do school‐based interventions aimed at improving language, literacy, and/or mathematical skills increase children's and adolescents' EFs?*

As a secondary objective, we will examine the following research question, if data allows it: *How are the effects of language, literacy, and mathematics interventions on EFs moderated by the subject of the intervention, child age or grade, the type of EF measured, and the at‐risk status of participants?*

We prespecify moderators regarding subject, child age, the type of EF measured, and the at‐risk status of participants, which corresponds to our confirmatory moderator analysis (see the Subgroup analysis and investigation of heterogeneity section for definitions of these variables). We will also examine the association between effect sizes and other study characteristics. As relevant study characteristics may be numerous, theoretically ambiguous, and difficult to prespecify, this analysis will be exploratory. That is, our third research questions is: *How are the effects of language, literacy, and mathematics interventions on EFs moderated by study characteristics?*

Lastly, our fourth research question is: *What are the effects of the included interventions on language, literacy, and mathematical skills?*

The fourth research question is motivated by the risk that the included interventions may be ineffective regarding their primary aim: to improve language, literacy, and mathematical skills. That is, if we find no effects on EFs, then ineffective interventions may be an explanation.

Examining the effects on language, literacy, and maths skills may also tell us something about the relationship between these skills and EF. For example, if we find effects on EFs despite finding no effects on language, literacy, and math skills, such results would suggest that the effects on EFs are less likely to be caused by interventions creating new cognitive routines related to language, literacy, and maths, which are also useful for solving EF tasks, or by improved domain‐specific knowledge. The effects on EFs would in that case be more likely to be caused by the interventions involving the use of EFs directly in the training of language, literacy, and maths.

As we require studies to have measured effects on EFs, it is important to note that the included interventions in this review are unlikely to be representative of language, literacy, and math interventions in general, and the effects we will estimate on these skills are similarly unlikely to be representative.

## 3 METHODS

### 3.1 Criteria for considering studies for this review

#### 3.1.1 Types of studies

We will include quantitative and experimental primary studies which examine the effects of school‐based intervention. Eligible studies must use a treatment‐control group design, such as RCTs, in which the assignment to treatment is determined by a random sequence, QRCTs, in which the assignment to treatment is determined by means such as alternate allocation, person's birth date, the date of the week or month, case number, or alphabetical order, and QESs, in which the assignment to treatment occurred, for example, in the course of usual decisions, by a (non‐random) researcher decision, or by a natural experiment (i.e., through some form of ‘natural’ or administrative process, which is outside the control of researchers).

Treatment‐control studies need to assign at least two ‘units’ (e.g., schools, classes, or students/children) to the treatment group and two units to the control group to be included. Treatment effects are difficult to separate from unit effects in studies with only one unit in either the treatment group or the control group. Effect sizes must also satisfy specific risk of bias criteria before contributing to the data synthesis (for these criteria, see the Assessment of risk of bias in included studies section). Studies in which all effect sizes are excluded from the data synthesis due to risk of bias criteria will still be included in the review.

Control groups might be defined as TAU conditions (including waiting list control groups), or a placebo intervention. Studies that only compares groups receiving different interventions, which are all hypothesised to improve academic or EF skills, will be excluded.

We will exclude non‐intervention studies, such as observational or descriptive studies, and qualitative study designs, as well as single‐subject before‐after designs, in which participants act as their own control group. Other reviews will not be included in this synthesis, although we will keep track of relevant reviews and use them where appropriate, for example, for citation tracking purposes.

Only studies published in English, German, Danish, Swedish and Norwegian are eligible, due to language restrictions in the review team.

#### 3.1.2 Types of participants

The eligible population samples for this review are children and adolescents attending either pre, primary, or secondary school (including high school). We will include both normally achieving, not‐at‐risk students as well as those identified as at‐risk because they are low performing or educationally disadvantaged. Furthermore, we will include clinical samples irrespective of the diagnosis, including samples of both physically and mentally disabled children. Whenever possible, we will record the status and/or diagnosis of children for later use in the analysis. We will not place a restriction on the type of preschool and school, that is, state, private, public, and boarding schools are all eligible for inclusion. Students can also attend both mainstream school or special education schools.

We will not include interventions performed in higher education, for example, at universities, or professional development programs. We will not include interventions performed outside of the school year or school day, that is, summer schools and after school programs are not included, unless the intervention has a vital component embedded in the normal school day setting.

#### 3.1.3 Types of interventions

The review will include primary studies examining language, literacy, and mathematical interventions carried out in preschool and school settings. Interventions might vary in duration and methodology and do not have to be prevalidated in terms of having an effect on language, literacy, or maths skills. However, interventions must constitute a condition different from TAU at the school or preschool. As TAU instruction may include components that improve EF skills without improving language, literacy, or maths skills, including such conditions would risk introducing a confounding element into the analysis.

As an example of what we mean with being different from treatment as usual at the school, Araujo et al. (2016) examined the effects of teacher and classroom quality on, amongst others, measures of EF, by using an assignment rule where children were assigned to classrooms in a manner that was close to random. Although this intervention trained many of the skills we are interested in, no child received a different instruction than treatment as usual in their school because of the intervention. The only aspect that differed was the ‘quality’ of the teacher and peers in the classroom, and this study will therefore not be included.

Interventions should be applied in a school or preschool setting and must be administered by teachers, teaching assistants, or the like. Thus, solely increasing the amount of homework or the amount of time parents are encouraged to train their children's skills at home, or in other types of family care, do not constitute an eligible intervention. Examples of such interventions are the Hanen parent programs *It Takes Two to Talk* ^*®*^ *—The Hanen Program* ^*®*^ *for Parents of Children with Language Delays and Target Word™*, or *The Hanen I'm Ready!™ Program for Building Early Literacy in the Home*.

Interventions must be aimed at enhancing students' academic achievement by training at least one specific mathematical, literacy or language skill. Interventions with explicit focus on only improving students' metacognitive strategies or study skills are not eligible for this review. To avoid confounding components, interventions with the specific purpose of training EFs are not eligible for this review. An example of such an intervention condition can be found in a study by Fuchs (2016); where one of the intervention groups received a reading comprehension program combined with working memory training, and one intervention group received only the reading comprehension program. The combined condition is not eligible for our review, whereas the condition in which the children only received reading comprehension training is eligible.

We recognise that the targeted academic skills most certainly will vary across pre, primary and secondary schooling. Therefore, we expect to include a range of different school‐based interventions targeting different specific skills in language, literacy, and maths domains. Below we present a range of skills that can be targeted in order for an intervention to be eligible. Please note that this typology can be subject to changes and developments throughout the review process, so the skills listed below should not be seen as an exhaustive list.

##### 3.1.3.1 Maths skills

Interventions in math which aimed at enhancing students' numeracy skills can focus on a variety of content domains. Specific aims of the interventions can thus be improving students' number sense, their understanding of shapes and geometry, or basic addition and subtraction. Fractions, probabilities and algebra are also relevant content domains. Examples of eligible intervention programs are *Big Math for Little Kids*, *Number Worlds*, and *Math Recovery*.

##### 3.1.3.2 Language and literacy skills

Interventions aimed at improving language and literacy skills can target for example vocabulary knowledge, accuracy of sound, consonant repertoire, decoding, phonemic awareness, phonological awareness, letter‐sound associations, or letter knowledge. We do not define learning a second or third language in school as a language intervention. Examples of eligible intervention programs are *Reading Recovery*, *Orton‐Gillingham*, *HELPS*, *Nuffield Early Language Intervention*, or *READ180*. On the one hand, language skills influence children's phonological awareness, which is a major component in the development of literacy skills (Anthony, 2005). On the other hand, improved reading skills may improve language skills (Morrison, 2019; Stanovich, 1986), and vocabulary interventions may affect language skills as well as for example reading comprehension (Hjetland, 2017; Rogde, 2019). We therefore expect substantial overlap in the targeted content domains among language and literacy interventions.

If an intervention applies multiple programs all targeting specific academic skills, it will be eligible as long as none of the programs target EF skills directly. For example, Weiland (2013) examines spillover effects on EF skills from a prekindergarten curriculum intervention. The curriculum intervention consists of the two programs *OWL—Opening the World of Learning* that targets literacy and language skills, and *Building Blocks* that targets math skills. Since neither of these programs in and of itself targets EF skills, the combined curriculum intervention of the two programs is eligible for inclusion.

#### 3.1.4 Types of outcome measures

We plan to include two types of outcome measures: measures of EFs (our primary outcome) and measures of academic achievement (our secondary outcome). We will include both end‐of‐intervention tests and follow‐up tests conducted after the end‐of‐intervention. We describe how we will analyse the outcomes with different measurement timing in the Data synthesis and Subgroup analysis and investigation of heterogeneity sections.

##### 3.1.4.1 Primary outcomes

The primary outcome of this review is measures of EFs in the child. We wish to include both direct measures obtained by use of cognitive tests such as *Delis‐Kaplan Executive Function System* (Delis, 2004) as well as indirect measures such as those from the different versions of the *Behaviour Rating Inventory of Executive Function* (e.g., Gioia, 1996). We wish to include studies of EFs in the broad sense, meaning that both measures of working memory, inhibitory control, and cognitive flexibility (attention shifting and control) are included. However, only studies applying prevalidated measures of executive functioning are eligible for inclusion. That is, the measures should be validated on other samples and not developed by researchers for the intervention at hand. Researcher‐developed measures risk inflating effect sizes (e.g., Slavin, 2011). Type of measure (indirect vs direct) are included as a possible moderator in our subgroup analyses and investigations of heterogeneity, to examine whether differences between indirect and direct measures exist in our data.

For a study to be eligible, we must be able to confirm that the applied (sub)test of EFs are in fact prevalidated. As a result, measures that we cannot positively confirm as prevalidated will be excluded. If a study applies a modified version of a prevalidated test of EFs, the study is also ineligible. This is motivated by the fact that EFs is a broad and often discussed ‘umbrella term’ referring to a number of different constructs. Especially, publications from previous decades might use the term EFs to refer to constructs that we do not consider relevant for the term today. Other reviewers, such as Jacob (2015), handled this issue by simply excluding all papers published prior 2000.

Working memory is a complex term referring to a series of coherent brain functions related to memory. However, as with EFs, the term is applied dissimilarly by different researchers, and especially the distinction between ‘working memory’ and ‘short‐term memory’ is disputed, as the two functions are closely related and somewhat overlapping. To account for the at times different use of ‘working memory’, we will also include studies using measures of short‐term memory as the outcome measure.

As an example of a study that will not be included, Goldstein (1976) provided perhaps the first test of whether a reading intervention affected memory skills and found positive effects on a test of short‐term sequential memory in a small‐scale trial. However, as the tests used by Goldstein (1976) were not prevalidated measures of EFs, this study will not be included. Another example, Stebbings (2020); used the Fluid reasoning subtest of Woodcock‐Johnson IV Tests of Cognitive Abilities. Although fluid reasoning is related to EFs, it is a more general concept (Diamond, 2013). We will use such tests as secondary outcomes but studies that only contain tests of fluid reasoning or other general cognitive skills tests will not be included in the review. That is, studies must include at least one primary outcome to be included.

##### 3.1.4.2 Secondary outcomes

Secondary outcomes include measures of academic achievement such as SAT scores and standardised measures of specific numeracy, literacy, and oral language skills. Furthermore, if available, additional cognitive measures, such as IQ, will be coded as a secondary outcome. Measures of secondary outcomes must be standardised to be included—that is, teacher/researcher‐developed tests are not eligible.

### 3.2 Search methods for identification of studies

Relevant studies will be identified through searches in electronic databases, governmental and grey literature repositories, hand search in specific targeted journals, citation tracking, contact to international experts, and internet search engines.

#### 3.2.1 Electronic searches

##### 3.2.1.1 Electronic databases

We plan to search the following databases:

- ERIC (EBSCO)
- PsycINFO (EBSCO)
- SocIndex (EBSCO)
- Academic Search (EBSCO)
- International Bibliography of the Social Sciences (ProQuest)
- Sociological Abstracts (ProQuest)
- Science Citation Index Expanded (Web Of Science)
- Social Sciences Citation Index (Web Of Science)

##### 3.2.1.2 Description and example of search‐string

The search string is based on the PICO(S)‐model. Using that model, we identified five aspects of the topic, and developed a search facet for each with relevant terms and synonyms. We do not intend to apply a time or language limitation on the database searches. All of the five facets will be searched as a title/abstract search. Some of the facets will also utilise the subject terms, which will vary according to each database thesaurus.

The search string includes a facet related to the outcomes (i.e., tests of EFs). We include this facet because pilot searches indicated that the number of hits would otherwise far exceed what the review team could handle, and included a very large proportion of irrelevant records. However, this choice creates a risk that we will miss relevant records that do not mention the outcome in the title or abstract, or are not indexed with the subject terms. For the following reasons, we believe that this risk is sufficiently low to motivate the restriction: First, it is typically costly to test EFs. For example, standardised tests of working memory often require that the tests are conducted by certified psychologists. Second, testing EFs is relatively unusal and therefore often an important part of a study. Thus, for these two reasons, researchers may be more likely to mention these outcomes in the abstract compared to other types of tests. Third, the electronic database search is not the only component of our search strategy. We will put extra efforts into backward and forward citation tracking, and into contacting authors of relevant studies and other experts. Thus, we should only miss studies that (a) we do not find in the database search, (b) that do not cite and are not cited by other relevant studies and reviews, and (c) are unknown to experts in the field.

An example of the search string as it will be implemented on the database ERIC is shown below.

- S23 is the final combination of the five facets
- S19‐22 covers the study design of the interventions
- S15‐18 covers the outcomes of the interventions
- S7‐14 covers the context/setting of the intervention
- S4‐6 covers the intervention type
- S1‐3 covers the population

**Table:** 

Search	Search Terms
S23	S3 AND S6 AND S14 AND S18 AND S22
S22	S19 OR S20 OR S21
S21	DE ‘Randomized Controlled Trials’ OR DE ‘Quasiexperimental Design’
S20	AB (trial* OR experiment* OR intervention* OR treatment* OR ‘control group*’ OR ‘compar* group*’ OR randomiz* OR randomis* OR ‘random‐assign*’ OR ‘random* assign* OR ‘quasi‐experiment*’ OR ‘quasiexperiment*’ OR ‘instrumental variable* OR ‘regression discontinuity’ OR ‘difference‐in‐difference*’ OR ‘differences‐in‐difference*’ OR ‘difference* in difference*’ OR ‘event stud*’ OR ‘event‐stud*’ OR matching OR ‘propensity score’ OR case‐control* OR ‘case control*’)
S19	TI (trial* OR experiment* OR intervention* OR treatment* OR ‘control group*’ OR ‘compar* group*’ OR randomiz* OR randomis* OR ‘random‐assign*’ OR ‘random* assign* OR ‘quasi‐experiment*’ OR ‘quasiexperiment*’ OR ‘instrumental variable* OR ‘regression discontinuity’ OR ‘difference‐in‐difference*’ OR ‘differences‐in‐difference*’ OR ‘difference* in difference*’ OR ‘event stud*’ OR ‘event‐stud*’ OR matching OR ‘propensity score’ OR case‐control* OR ‘case control*’)
S18	S15 OR S16 OR S17
S17	DE (‘Executive Function’ OR ‘Inhibition’ OR ‘Short Term Memory’ OR ‘Self Management’ OR ‘Attention’ OR ‘Concept Formation’ OR ‘Goal Orientation’)
S16	AB (‘executive function*’ OR ‘impulse control’ OR ‘emotion* control’ OR ‘emotion* regulat*’ OR ‘flexible thinking’ OR ‘working memory’ OR ‘working‐memory’ OR ‘self monitor*’ OR self‐monitor* OR ‘task initiation’ OR ((‘problem solving’ OR problem‐solving) AND (task OR test OR score)) OR ‘concept formation’ OR ‘goal orientation’ OR goal‐orientation OR (planning* AND prioriti* AND (organis* OR organiz*)) OR ‘self regulat*’ OR self‐regulat* OR attention* OR inhibition* OR ‘inhibitory control’ OR ‘short‐term memory’ OR ‘short term memory’ OR ‘immediate memory’ OR ‘verbal memory’ OR ‘nonverbal memory’ OR ‘delayed recall’ OR delayed‐recall OR ‘free‐recall’ OR ‘free recall’ OR ‘serial‐recall’ OR ‘serial recall’ OR ‘associative recall’ OR associative‐recall OR cogniti* flex* OR cogniti* refl* OR cogniti* control OR shifting OR set‐shifting OR ‘effortful control’ OR ‘self‐control’ OR ‘self control’ OR ‘adaptable thinking’ OR ‘task switch* OR ‘self management’ OR self‐management)
S15	TI (‘executive function*’ OR ‘impulse control’ OR ‘emotion* control’ OR ‘emotion* regulat*’ OR ‘flexible thinking’ OR ‘working memory’ OR ‘working‐memory’ OR ‘self monitor*’ OR self‐monitor* OR ‘task initiation’ OR ((‘problem solving’ OR problem‐solving) AND (task OR test OR score)) OR ‘concept formation’ OR ‘goal orientation’ OR goal‐orientation OR (planning* AND prioriti* AND (organis* OR organiz*)) OR ‘self regulat*’ OR self‐regulat* OR attention* OR inhibition* OR ‘inhibitory control’ OR ‘short‐term memory’ OR ‘short term memory’ OR ‘immediate memory’ OR ‘verbal memory’ OR ‘nonverbal memory’ OR ‘delayed recall’ OR delayed‐recall OR ‘free‐recall’ OR ‘free recall’ OR ‘serial‐recall’ OR ‘serial recall’ OR ‘associative recall’ OR associative‐recall OR cogniti* flex* OR cogniti* refl* OR cogniti* control OR shifting OR set‐shifting OR ‘effortful control’ OR ‘self‐control’ OR ‘self control’ OR ‘adaptable thinking’ OR ‘task switch* OR ‘self management’ OR self‐management)
S14	S7 OR S8 OR S9 OR S10 OR S11 OR S12 OR S13
S13	DE (‘Early Childhood Education’ OR ‘Kindergarten’ OR ‘Preschool Education’ OR ‘Preschools’ OR ‘Primary Education’ OR ‘Elementary Education’ OR ‘Elementary Schools’ OR ‘Secondary Education’ OR ‘Secondary Schools’ OR ‘Grade 1’ OR ‘Grade 2’ OR ‘Grade 3’ OR ‘Grade 4’ OR ‘Grade 5’ OR ‘Grade 6’ OR ‘Grade 7’ OR ‘Grade 8’ OR ‘Grade 9’ OR ‘Grade 10’ OR ‘Grade 11’ OR ‘Grade 12’)
S12	AB childhood N1 (education OR program* OR care OR initiativ* OR development*)
S11	TI childhood N1 (education OR program* OR care OR initiativ* OR development*)
S10	AB (‘primary education’ OR ‘secondary education’ OR school* OR preschool* OR pre‐school* OR kindergart* OR childcare OR ‘child* care’ OR daycare OR ‘day care’ OR pre‐primar* OR ‘pre primar*’ OR ‘early education’ OR pre‐K OR ‘pre K’ OR prekindergart* OR pre‐kindergart* OR nurser* OR ‘reception class’)
S9	TI (‘primary education’ OR ‘secondary education’ OR school* OR preschool* OR pre‐school* OR kindergart* OR childcare OR ‘child* care’ OR daycare OR ‘day care’ OR pre‐primar* OR ‘pre primar*’ OR ‘early education’ OR pre‐K OR ‘pre K’ OR prekindergart* OR pre‐kindergart* OR nurser* OR ‘reception class’)
S8	AB((grade* N1 (1 OR 2 OR 3 OR 4 OR 5 OR 6 OR 7 OR 8 OR 9 OR 10 OR 11 OR 12)) OR ((first OR second OR third OR fourth OR fifth OR sixth OR seventh OR eighth OR ninth OR tenth OR eleventh OR twelfth) N1 grade*))
S7	TI ((grade* N1 (1 OR 2 OR 3 OR 4 OR 5 OR 6 OR 7 OR 8 OR 9 OR 10 OR 11 OR 12)) OR ((first OR second OR third OR fourth OR fifth OR sixth OR seventh OR eighth OR ninth OR tenth OR eleventh OR twelfth) N1 grade*))
S6	S4 OR S5
S5	AB (reading* OR math* OR languag* OR literac* OR numerac* OR number* OR geometr* OR algebra* OR fraction* OR operation* OR arithmetic* OR addition* OR subtraction* OR multiplication* OR division* OR statistics* OR probability* OR calculus* OR combinatoric* OR computation* OR calculation* OR counting* OR ‘word problem*’ OR ‘word‐problem*’ OR measurement* OR comprehension* OR decod* OR ‘word identification*’ OR ‘word‐identification’ OR fluency OR phonic* OR ‘phon* aware*’ OR phonem* OR fluency OR spelling OR vocabulary OR alphabetic* OR letter* ‘print aware*’ OR ‘sound discrim*’ OR ‘rhyme detect*’ OR blending OR segmentation OR grammar OR syntax OR syntactic OR morpholog*)
S4	TI (reading* OR math* OR languag* OR literac* OR numerac* OR number* OR geometr* OR algebra* OR fraction* OR operation* OR arithmetic* OR addition* OR subtraction* OR multiplication* OR division* OR statistics* OR probability* OR calculus* OR combinatoric* OR computation* OR calculation* OR counting* OR ‘word problem*’ OR ‘word‐problem*’ OR measurement* OR comprehension* OR decod* OR ‘word identification*’ OR ‘word‐identification’ OR fluency OR phonic* OR ‘phon* aware*’ OR phonem* OR fluency OR spelling OR vocabulary OR alphabetic* OR letter* ‘print aware*’ OR ‘sound discrim*’ OR ‘rhyme detect*’ OR blending OR segmentation OR grammar OR syntax OR syntactic OR morpholog*)
S3	S1 OR S2
S2	AB (student* OR pupil* OR child* OR toddler* OR youth* OR adolescen* OR teenage* OR young*)
S1	TI (student* OR pupil* OR child* OR toddler* OR youth* OR adolescen* OR teenage* OR young*)

#### 3.2.2 Searching other resources

##### 3.2.2.1 Hand search

We believe journals covering the intersection between education and psychology are most likely to include studies related to our review topic. The chosen journals furthermore focus on different parts of the age range of children we will include. To ensure we identify the most recent references, we will hand search the following journals manually:

- *Journal of Educational Psychology*
- *Child Development*
- *Contemporary Educational Psychology*
- *Early Childhood Research Quarterly*
- *American Educational Research Journal*

This list is subject to change. We will search the journals going 5 years back, that is, from 2017 to 2022. The final list of hand‐searched journals will be documented in the review.

##### 3.2.2.2 Searches for unpublished literature in general

We have split the search strategies in sub‐sections for each type of unpublished literature. In general, most of the resources searched for this purpose include multiple types of literature and references. As an example, the resources listed to identify reports from national bibliographical resources also include working papers and dissertations, as well as peer‐reviewed references. A resource might be searched for multiple purposes, but for the sake of simplicity, it is only listed once as a resource.

##### 3.2.2.3 Search for dissertations

We will search the following resources for dissertations:

- ProQuest Dissertations & Theses Global (ProQuest)
- EBSCO Open Dissertations (EBSCO‐host)

Further resources for identifying dissertations might be added during the search process. A final list of resources will be included in the appendix of the review.

##### 3.2.2.4 Search for working papers/conference proceedings

We will search the following resources for working papers/conference proceedings:

- European Educational Research Association (EERA)—https://eera-ecer.de/
- American Educational Research Association (AERA)—https://www.aera.net/
- PsyArXiv—https://psyarxiv.com/
- Open Grey—http://www.opengrey.eu/
- Google Scholar—https://scholar.google.com/
- Google searches—https://www.google.com/
- Social Science Research Network—https://www.ssrn.com/index.cfm/en/[1]

Further resources for identifying working papers and conference proceedings might be added during the search process. A final list of resources will be included in the appendix of the review.

##### 3.2.2.5 Search for reports and non‐US literature

We will search the following resources for reports and *non‐US literature*:

- Danish National Research Database—http://www.forskningsdatabasen.dk/en
- SwePub—Academic publications at Swedish universities—http://swepub.kb.se/
- NORA—Norwegian Open Research Archives—http://nora.openaccess.no/
- CORE—research outputs from international repositories—https://core.ac.uk/
- Best Evidence Encyclopedia—http://www.bestevidence.org/[2]

Further resources for identifying reports might be added during the search process. A final list of resources will be included in the appendix of the review.

##### 3.2.2.6 Search for systematic reviews

We developed a specific search string to identify other systematic reviews in the databases listed above. This was done simultaneously with the development of the search‐string described above, and the identified relevant reviews are considered in the content of this protocol.

We will also search for systematic reviews on the following resources:

- Campbell Journal of Systematic Reviews—https://campbellcollaboration.org/
- Cochrane Library—https://www.cochranelibrary.com/
- Centre for Reviews and Dissemination Databases—https://www.crd.york.ac.uk/CRDWeb/

##### 3.2.2.7 Citation‐tracking

We will use citation‐tracking methods to identify more relevant literature. We will citation‐track all included records and all relevant reviews (e.g., those mentioned in section Why it is important to do this review and those found in other parts of our search) forwards by using Google Scholar and Web of Science and backwards by screening the reference lists.

##### 3.2.2.8 Contact to experts

We will contact international experts to identify unpublished and ongoing studies, and provide them with the inclusion criteria for the review along with the list of included studies, asking for any other published, unpublished or ongoing studies relevant for the review. We will primarily contact corresponding authors of the included studies found using the other sources mentioned above, and authors of relevant reviews.

### 3.3 Data collection and analysis

#### 3.3.1 Selection of studies

The screening process for identifying relevant studies is split in two overall stages: (1) screening based on title and abstract, and (2) screening based on full text. To ensure the quality of the screening process and reduce potential errors, we make use of independent double screening at both stages (Polanin, 2019; Stoll, 2019). The screeners are blinded to each other's work until comparing final judgements of the screened references. If the two screeners cannot agree on the inclusion/exclusion of a specific reference, then this reference is sent to one of the review authors for final judgement.

We will conduct a pilot screening for each overall screening stage and for each screener. In the pilot screening based on title and abstract, the review team will screen and compare 80–100 references. The review team will then discuss and resolve potential disagreements and uncertainties regarding the eligibility criteria. If the interrater agreement is above 90% in the pilot screening, then the rest of the references will be screened. If the interrater agreement is below 90% in the first pilot, the review team members will perform a second pilot screening to ensure sufficient reliability before the rest of the references are screened. At the full text stage of the screening process, the pilot will consist of 8–10 studies. The pilot procedure at second level is otherwise identical to the process described for first level. The review team will meet with regular intervals in all stages of the screening process to discuss uncertainties and minimise ‘coders drift’ (Polanin, 2019). The screening tool and guidance questions for screeners can be found in Supporting Information: Appendix 1. Potential changes to the tool will be discussed during the pilots for each stage.

We will use the machine learning (ML) functionality in EPPI Reviewer 4 to conduct priority screening in the title and abstract screening phase. We will first screen 1000 records on title and abstract using independent double screening. We will then rank the remaining records by the ML algorithm's probability of a record being included, and screen those with the highest probability first. We intend to screen in batches of 1000 and re‐rank the remaining records after each batch has been completed.

Using priority screening has a dual purpose. First, we may find relevant records earlier in the screening process, which would mean that we can start the coding and the risk of bias assessment earlier and thereby speed up the completion of the review. Second, despite including a facet covering outcomes, our pilot searches indicate that we may find a large number of records. Should we find more than 10,000 unique records in the electronic database searches, we will consider switching to single person screening if the ratio of included to excluded records becomes very low (e.g., less than 1/100) and substantially lower than in the batches screened first (otherwise, the priority ranking may not be good enough). If we switch to single person screening, we will double screen a random sample of 10% of each batch to check that we do not miss relevant records.

We will present the overall search and screening process in a flow chart in the final review.

During the screening process, none of the review authors or review team members will be blind to the authors, journals, or institutions responsible for the publication of eligible studies.

#### 3.3.2 Data extraction and management

Two members of the review team will independently extract and code data from the included studies. The coding tool will be piloted and is subject to potential revisions throughout the coding process. See Supporting Information: Appendix 2 for the first and current version of the tool. From all included studies, we extract data on publication characteristics, study characteristics, participant characteristics, intervention characteristics, and outcome characteristics. If any disagreement or uncertainty emerges during the data extraction process, a third reviewer (most often another of the review authors) with the appropriate expertise will be consulted.

All extracted data will be stored electronically using EPPI Reviewer 4 and Microsoft Excel.

#### 3.3.3 Assessment of risk of bias in included studies

Two members of the review team, and always at least one of the review authors, will independently assess the risk of bias for each eligible study outcome. The review team members will discuss disagreements in their ratings, and if necessary, another review author will be contacted for final agreement. We will report the agreed risk of bias assessments for all included studies in the final review.

For included non‐randomised studies (QRCT and QES), we assess the risk of bias for all included outcomes applying Cochrane's ROBINS‐I tool (Sterne, 2016). For all included randomised studies, we assess the risk of bias of all outcome measures using the revised version of Cochrane's risk of bias tool, ROB‐2 (Elridge, 2016; Sterne, 2019). In this section, we briefly outline the characteristics of each tool.

##### 3.3.3.1 ROBINS‐I

The ROBINS‐I tool covers seven domains. These seven domains broadly cover types of biases, which might be introduced into non‐randomised trials. The domains in ROBINS‐I are:

1. Confounding bias
2. Selection bias
3. Classification bias
4. Deviation bias
5. Missing data
6. Measurement bias
7. Reporting bias

In ROBINS‐I, every outcome measure is rated on each domain as either having a ‘low’, ‘moderate’, ‘serious’, or ‘critical’ level of bias. In cases without sufficient evidence for rating the bias level, the outcome gets a rating of ‘no information’. If a study outcome receives a ‘critical' rating on at least one domain, it is considered too biased to provide useful evidence on the effects of the intervention. As a consequence, the outcome is excluded from the data synthesis. We will not continue the risk of bias assessment of an outcome measure if a domain is rated ‘critical’.

##### 3.3.3.2 ROB‐2

The five domains in ROB‐2 cover types of biases potentially influencing the results found in RCTs. These are:

1. Bias arising from the randomisation process (preintervention)
2. Bias arising from deviations from the intervention
3. Bias arising from missing outcome data
4. Bias arising from the measurement of outcomes
5. Bias arising from the selection of reported results

In each domain of the ROB‐2 tool, every outcome measure is rated as either calling for ‘low’, ‘some’, or ‘high’ concerns.

In both tools, an overall rating may be made on the basis of the domain ratings. A rating of ‘some concerns’ in multiple domains of the ROB‐2 assessment tool may lead to a decision of an overall judgement of ‘high’ risk of bias for that outcome. A ‘serious’ risk of bias in multiple domains of the ROBINS‐I assessment tool may lead to a decision of an overall judgement of ‘critical’ risk of bias for that outcome, and it will be excluded from the data synthesis. Outcome measures which have been excluded due to multiple ratings of ‘serious’ in individual domains will be listed in the final review, along with reasons for exclusion. The overall rating of the study also contains an assessment of the overall bias direction for the assessed outcomes. A further commonality is that both tools require prespecification of the effect type that will be assessed. We are most interested in, and believe that most studies will report estimates that are closer to, the effect of starting and adhering to the intervention than the effect of assignment to the intervention.

In the case of an RCT, where there is evidence that the randomisation has gone wrong or is no longer valid, we will assess the risk of bias of the outcome measures using ROBINS‐I instead of ROB‐2. Examples of reasons for assessing RCTs as non‐randomised studies may include studies showing large and systematic differences between treatment conditions while not explaining the randomisation procedure adequately; studies with large‐scale differential attrition between conditions in the sample used to estimate the effects; or studies selectively reporting results for some part of the sample or for only some of the measured outcomes. In such cases, differences between the treatment and control conditions are likely systematically related to other factors than the intervention and the random assignment is, on its own, unlikely to produce unbiased estimates of the intervention effects. As ROBINS‐I allows for an assessment of for example confounding, we believe it is more appropriate to assess effect sizes from studies with invalid randomisation using ROBINS‐I than ROB‐2. If so, we will report this decision as part of the risk of bias assessment of the outcome measure in question. As other effect sizes assessed with ROBINS‐I, these effect sizes may receive a ‘Critical’ rating and thus be excluded from the data synthesis.

##### 3.3.3.3 Definition of critical confounders

ROBINS‐I dictates that reviewers should define critical confounders relevant to most or all eligible studies at the protocol stage. In the case of this review, we define the critical confounders as *performance at baseline* and *age*. Other important confounders may be for example the students' socioeconomic status and gender. If other confounders are unbalanced between the treatment and control group, or the comparison groups, the lack of balance will be reflected in a higher rating (i.e., defining critical confounders does not imply that other confounders will not be considered). However, we anticipate that confounding from for example socioeconomic status and gender will often be captured by performance at baseline.

Confounding happens when prognostic factors determine the allocation of participants into treatment conditions. Uncontrolled confounding will bring about systematic differences between the experimental conditions and thus compromises comparability. Confounding factors can be observable (e.g., age) or unobservable to the researcher (e.g., personal motivation). Inherently, unobservable confounding factors are harder for researchers to examine and control than observable confounding factors.

Performance at baseline is generally considered a strong prognostic factor in relation to posttest outcomes in preschool and school interventions (e.g., Hedges, 2007). Furthermore, performance at baseline is likely to capture the effects of many other important determinants of performance. For example, if gender differences between the treatment and control group are present at baseline, and gender is an important influence on test scores, these differences should to a large extent be reflected in the pretest scores. Furthermore, prognostic factors like socioeconomic status are slow‐changing variables, which means that any posttest differences between the treatment and control group across such variables should also be reflected in the pretest scores (e.g., the proportion of girls and of low SES students were, e.g., not associated with effect sizes in the moderator analyses in Dietrichson, 2017; Dietrichson, 2020; Dietrichson, 2021).

Although performance at baseline could, in most cases, be expected to also capture age differences between the treatment and control group, the skills we examine may develop fast, especially when children are young, and we therefore believe the age of the participants is a second critical confounder.

#### 3.3.4 Measures of treatment effect

In our main analysis, we aim to compare the intervention condition with the control condition on measures of EF, and secondary on measures of academic achievement.

We expect that almost all studies found in this literature use continuous outcome measures. For continuous data, we will calculate the standardised mean difference (SMD) where possible, since our outcomes (EFs and academic achievement) are measured and reported with a wide range of different scales. To correct for upward bias in small samples, we will use the small sample bias‐corrected Hedges' *g* in our analysis (Borenstein, 2009; Hedges, 1981; Lipsey, 2001). Hedges' *g* and its standard error are calculated as (Lipsey, 2001, pp. 47–49):

(1) $$ES_{g}=[1-(3/(4N-9))]\times [(X_{1}-X_{2})/s_{p}],$$

(2) $$SE_{g}=\sqrt{\left[\left(N/(n_{1}n_{2}))+(ES_{g}^{2}/2N\right)\right]},$$

where *N = n*_1_ +* n*_2_ is the total sample size, *X* is the mean in each group, and *s_p_* is the pooled standard deviation defined as

(3) $$s_{p}=\sqrt{\left[\left((n_{1}-1)s_{1}^{2}+(n_{2}-1)s_{2}^{2})/(n_{1}+n_{2}-2\right)\right]}.$$

Here, *s*_1_ and *s*_2_ denote the unadjusted standard deviations of the treatment and control group. We will record both post and pretest standard deviations if available. For our main analysis, we will calculate SMD's with posttest standard deviations, as these values are more likely to be consistently reported in this area of research and due to the possibility of floor effects at pretest. Only when posttest standard deviations are not available, but pretests standard deviations are, we intend to use pretest standard deviations as replacements to avoid missing values. However, we do recognise that using pretest standard deviations can be advantageous, due to the possibility of the treatment affecting variability (Lipsey & Wilson, 2001). Thus, if we detect a difference between pre and posttest standard deviations, we will check the sensitivity of our calculated SMD's—for further details see our *Sensitivity analysis* section.

We will use covariate adjusted means whenever available. We will use treatment‐on‐the‐treated (TOT) or local average treatment effects (LATE) whenever possible, and test whether results are sensitive to the inclusion of intention‐to‐treat (ITT) estimates of the effects. If there is a mix of studies with some reporting change scores and others reporting final values, we will contact the trial investigators and request the final values. If these are unobtainable, we will also provide a separate analysis of change scores and final values.

If included studies report dichotomous outcome data, we will use the methods described in Sánchez‐Meca (2003); specifically the Cox transformation, to transform the outcome data into SMDs.

#### 3.3.5 Unit of analysis issues

Errors in statistical analysis can occur when the unit of allocation differs from the unit of analysis. In cluster‐randomised trials, participants are randomised to treatment and control groups in clusters, for example, localities or schools. QES may also include clustered assignment of treatment. Effect sizes and standard errors from such studies may be biased if the unit‐of‐analysis is the individual and an appropriate cluster adjustment is not used (Higgins, 2011).

If possible, we will adjust effect sizes individually using the methods suggested by Hedges (2007) and information about the intra‐cluster correlation coefficient (ICC), realised cluster sizes, and/or estimates of the within and between variances of clusters. If it is not possible to obtain this information consistently across included studies, we will adjust the effect sizes using estimates from the literature of the ICC in Hedges (2007), and assume equal cluster sizes in the treatment and control group. We will use an ICC of 0.11, which approximately corresponds to the average of ICCs taken over grades from kindergarten to grade 12 and maths and reading tests in Hedges (2007; reported in tab. 2 and 3, pp. 68–69, models with covariates). We will test if our results are sensitive to this choice by using ICCs of 0 (the theoretical minimum) and 0.32 (the empirical maximum in the same two tables). We are not aware of similar evidence of typical ICCs for studies conducted in preschool, and we will use these ICCs also for preschool studies (i.e., if they cannot be adjusted individually). To calculate an average cluster size, we will divide the total sample size in a study by the number of clusters (typically the number of classrooms or schools).

In some cases, several studies may have used the same sample of data, for example, studies using the same administrative data. We will review all such studies, but will only include one estimate of the effect from each sample of data in the meta‐analysis to avoid duplication. The choice of which estimates to include will be based on our risk of bias assessments. We will choose the estimates that we judge to have the least risk of bias.

We expect that studies will report multiple and dependent effect sizes. Dependencies between effect sizes may for example arise because the same children are tested on multiple tests, because studies contain multiple treatment groups, or include multiple interventions per individual. Instead of only using one effect size per study, we intend to apply robust variance estimation (RVE) methods in our data synthesis and analysis (e.g., Hedges, 2010; Tanner‐Smith, 2016; Tipton, 2015; Tipton, 2015). In particular, we will use the correlated‐hierarchical effect (CHE) framework developed by Pustejovsky (2021; described in the Data synthesis section).

#### 3.3.6 Dealing with missing data

Studies must permit calculation of a numeric effect size for the outcomes to be eligible for inclusion in the meta‐analysis. Where studies have missing summary data, such as missing standard deviations or means, we will derive these where possible from, for example, *F*‐ratios, *t*‐values, *χ* ^2^ values and correlation coefficients using the methods suggested by Lipsey (2001). If these statistics are also missing, the review authors will request information from the study investigators. If missing summary data necessary for the calculation of effect sizes cannot be derived or retrieved, the study results will be reported in as much detail as possible, i.e., the study will be included in the review but excluded from the meta‐analysis. Missing data and attrition rates for the individual studies are assessed with the risk of bias tools, where both ROB‐2 and ROBINS‐I have specific domains focusing on biases arising from missing data (Sterne, 2016; Sterne, 2019).

#### 3.3.7 Assessment of heterogeneity

Heterogeneity can stem from either an expected variation in effects or from sampling errors in included studies. In this review, we assume that variation in effects will occur and will therefore use a random‐effects model in our main analysis (see also the Data synthesis section). Consequently, we expect to find heterogeneity in our analyses. We aim to assess the level of heterogeneity with the *Q* and the *I* ^2^ statistics, and the within (*ω* ^2^) and between‐study variance (*τ* ^2^) (Higgins, 2003; Pustejovsky, 2021), as well as prediction intervals (defined below).

We will report prediction intervals to examine and show how effects are dispersed. Prediction intervals are based on the mean effect size and the standard deviation of effect sizes, instead of standard errors, which are used in the calculation of confidence intervals. We will calculate prediction intervals wherein effects will lie 95% of the time. Since the mean and the standard deviation can only be estimated with some error, we calculate the lower and upper limits of the prediction intervals with the modifications provided in formulas 4 and 5 (Borenstein, 2017; Higgins, 2009):

(4) $$LL_{pred}=ES_{g}-t_{df}\times \sqrt{\left(\tau^{2}+V_{g}\right)},$$

(5) $$UL_{pred}=ES_{g}+t_{df}\times \sqrt{\left(\tau^{2}+V_{g}\right)},$$

where *ES*_*g*_ is the estimated mean effect size, *t*_*df*_ is the critical *t*‐value for our degrees of freedom, *τ* ^2^ is the estimated between‐study variance, and *V*_*g*_ is the variance of the mean effect size.

#### 3.3.8 Assessment of reporting biases

Reporting bias might refer to both publication bias and selective reporting of outcome data and results. Bias from selective reporting of outcome data and results are assessed in both ROB‐2 and ROBINS‐I.

We intend to use the following methods to assess the extent of publication bias. First, we will show funnel plots and examine whether they are asymmetric (Higgins, 2011). To formally test for asymmetry, we will use a version of Egger's test (Egger, 1997) suggested by Rodgers (2021). Egger's test examines asymmetry by including a measure of effect size precision as a predictor in a meta‐regression with effect sizes as the outcome variable. A significant coefficient on the precision measure is interpreted as evidence of asymmetry. However, Pustejovsky (2019) showed that the original Egger's test often rejects the null hypothesis of no asymmetry at higher rates than the chosen level of statistical significance (i.e., the Type I errors were inflated). Rodgers (2021) examined a version of Egger's test, which handled effect size dependence within studies by using RVE. In their simulations, this ‘Egger Sandwich' test had better properties in terms of Type I errors than the original Egger's test, and other tested methods. As Rodgers (2021), we will interpret the rejection of the null hypothesis of no asymmetry in a one‐sided test with significance level 0.05 as an indication of asymmetry.

Asymmetric funnel plots are not necessarily caused by publication bias (and publication bias does not necessarily cause asymmetry in a funnel plot). If asymmetry is present, we will consider possible reasons for the asymmetry and test how sensitive our results are to publication bias using the method developed by Mathur (2020). Furthermore, Egger's test, in both the regular and 'Sandwich' version, has a limited capacity to detect publication bias when the number of included studies is small (Egger, 1997; Rodgers, 2021), which may be the case in our review. As different methods may yield different results, we will, if the number of studies permit it, consider using selection models (e.g., Andrews, 2019; Hedges, 1992; Hedges, 2005), which may identify and correct for the presence of publication bias.

#### 3.3.9 Data synthesis

The data synthesis will be conducted in the following steps: First, we will provide descriptive summaries of the contextual, methodological, and outcome characteristics for the studies included in the data synthesis. Second, our main effects analysis will report a weighted average effect size comparing the results on EF skills tests of children in the intervention groups with children in the control groups (corresponding to our first research question). As a secondary measure, we will examine the effects on tests of academic achievement (corresponding to our fourth research question). Along with the main analysis, we will present forest plots, prediction intervals, and heterogeneity statistics. Third, as far as our data permit, we will conduct our proposed moderator and sensitivity analyses (described in the Subgroup analysis and investigation of heterogeneity section). We intend to perform all statistical analyses in R.

In all our analyses, we assume a random‐effects model. We will use inverse‐variance‐based weights. To estimate the overall effect size and heterogeneity statistics, we will use the RVE methods developed by Pustejovsky (2021). This method will allow us to take into account both dependencies between effect sizes that arise because the same sample is tested on different tests (‘correlated effects') and because different samples are included in the same study (‘hierarchical effects’). As both these types of dependencies are conceivable in our case, this feature is an advantage over the original RVE method developed by Hedges (2010). The original RVE procedure may furthermore have some disadvantages in terms of estimating heterogeneity parameters (see Tanner‐Smith, 2016 for a discussion). The CHE method is implemented in three steps.

In Step 1, we identify an appropriate working model based on the features of our sample (e.g., whether there are correlated or hierarchical effects, or both). A baseline value for the correlation between pairs of effect sizes from the same study (*ρ*) has to be specified. We will choose 0.6, as suggested by Pustejovsky (2021); but test if our results are sensitive to lower (0.4) and higher (0.9) values. We chose the latter value because some of the results in Pustejovsky (2021) were sensitive to using values of *ρ* higher than 0.8.

In Step 2, based on the chosen working model, we will estimate meta‐regressions using a combination of the *clubSandwich* (Pustejovsky 2021) and *metafor* (Viechtbauer, 2010) packages in R. In the main effects analysis, the dependent variable is either tests of EF skills, or academic achievement. If all outcomes have the same measurement timing (e.g., are measured at or close to the end of intervention), we will regress the dependent variable on an intercept only, which provides the weighted average effect size. If the outcomes are measured with different timing, we will include an indicator for the follow‐up outcomes. We will use the *clubSandwich* package to specify the correlation structure between effect size estimates within studies. Then, we will estimate the random‐effects variance components, inverse‐variance weight matrices, and the meta‐regression coefficients using the restricted maximum likelihood (REML) procedure in the *metafor* package.

In step 3, we will calculate confidence intervals based on the RVE standard errors obtained from the *clubSandwich* package. These standard errors are adjusted for small‐sample bias as suggested by Tipton (2015) and Tipton and Pustejovsky (2015). We intend to report 95% confidence intervals for all analyses. As the results in Tanner‐Smith (2013) and Tipton (2015) suggest that standard errors from the RVE procedure are unreliable when the adjusted degrees of freedom are below 4, we will report when the degrees of freedom are below or close to 4.

#### 3.3.10 Subgroup analysis and investigation of heterogeneity

We intend to conduct a moderator analysis to identify the characteristics of interventions, participants, outcome measures, and study characteristics that are possibly associated with smaller and larger effects on the EF outcomes (i.e., we will not conduct a moderator analysis for our secondary outcome). For the moderator analysis, we will use the same type of mixed‐model meta‐regression and RVE procedure as in our main effects analysis and report 95% confidence intervals for all regression parameters. If we find outcomes measured at end‐of‐intervention and longer follow‐ups, all moderator analyses will include an indicator for follow‐up tests.

In our confirmatory moderator analysis, corresponding to our third research question, we will pool all effect sizes and then sequentially add the following moderators:

- Indicators for intervention content domain (contrasting math interventions with literacy and language interventions).
- Indicators for school setting and age (contrasting preschool with primary and secondary school).
- Indicators for EF outcome measures (contrasting measures of working and short‐term memory, inhibition, cognitive flexibility, and composite/broader constructs, such as self‐regulation).
- An indicator for at‐risk target groups (contrasting at‐risk with not‐at‐risk groups).

As Clements (2016) suggested, math may have a more direct connection to EFs and math interventions may therefore have larger effect sizes. We will pool literacy and language interventions due to the overlap discussed in the Types of interventionssection, and to improve statistical power. The discussion in the How the intervention might work section indicated that the neural systems of younger children are more plastic. Effect sizes may therefore be larger for earlier interventions than later interventions. Although the direction was not clear from our theoretical discussion (e.g., because we do not know which language, literacy, and math skills transfer to what EF skill), the type of EF skill measure may moderate effect sizes. Peng (2020) found a weaker bidirectional relationship between EF skills and academic skills for at‐risk than for not‐at‐risk students, which is why we want to examine this contrast.

As moderators may be correlated, we prefer to include all variables in one regression. However, as it decreases the degrees of freedom, adding all moderators simultaneously may not be feasible. If this is the case, we will prioritise moderators where we have complete information and then in the order mentioned above. That is, we will first add indicators of the intervention's content domain, then the school setting/age, the type of EFs, and lastly the at‐risk indicator, stopping when we risk not being able to reliably estimate a previously added indicator (i.e., when the adjusted degrees of freedom is below 4).

Study and participant characteristics often moderate the effect sizes of school interventions (e.g., Cheung, 2016). However, as we may not find enough studies to include all potentially relevant study characteristics in one regression and the direction of the influence on effect sizes of these moderators is theoretically ambiguous, we consider the examination of study characteristics exploratory. If we find that including study characteristics in the meta‐regressions change the results for the indicators related to intervention content, school setting, EF outcome measure, or the at‐risk indicator, we will take this into account in our conclusions. Furthermore, our discussion about how the interventions may work suggested that there may be interaction effects (e.g., between the content of the interventions and the age of the participants). If data permits, we will include an exploratory analysis of such interactions.

The exact definition of moderators may be subject to change during the data extraction process. However, a preliminary version of the codebook, including more details on some of the moderators can be found in Supporting Information: Appendix 2.

### 3.4 Sensitivity analysis

To explore the sensitivity of our results, we intend to perform the following sensitivity analyses: Examination of distribution of effect sizes, examination of our calculated SMDs, examination of methodological quality, and publication bias (the last sensitivity analysis is described in the Assessment of reporting biases‐section).

#### 3.4.1 Distribution of effect sizes

We will examine the distributions of effect sizes for each outcome category for the presence of outliers. If outliers are found, we will examine the sensitivity of the results by Winsorising the outliers to the nearest non‐outlier value (e.g., Lipsey, 2001).

#### 3.4.2 Calculated standardised mean differences

We intend to estimate SMDs with posttest standard deviations as these values are more likely to be reported. If we find differences between pre and posttest standard deviations, we will then check the sensitivity of our results by calculating alternative SMDs where the pretest standard deviations are used in the calculation of SMDs.

We will also examine whether or not possible differences in baseline between the treatment and control group affects our results. If we find that such differences exist, we will calculate alternative SMDs taking into account these baseline differences.

#### 3.4.3 Methodological quality

To examine methodological quality, we will consider sensitivity analyses for each domain in the risk of bias assessments. The studies which have received a rating of either ‘high’ or ‘serious’ in a domain will be removed from the model to test for sensitivity in models’ results.

## CONTRIBUTIONS OF AUTHORS

The lead author of the review is Jens Dietrichson. The co‐authors of the review are Elizabeth Bengtsen, Julie Kaas Seerup, Martin Williams Strandby, Morten Kjær Thomsen, and Bjørn Viinholt. The responsibilities of the authors are given below:

- Content: Jens Dietrichson, Julie Kaas Seerup, Martin Williams Strandby, and Morten Kjær Thomsen.
- Systematic review methods: Jens Dietrichson, Julie Kaas Seerup, and Morten Kjær Thomsen.
- Statistical analysis: Jens Dietrichson and Julie Kaas Seerup.
- Information retrieval: Elizabeth Bengtsen and Bjørn Viinholt

## DECLARATIONS OF INTEREST

Two of the review authors (Dietrichson, Seerup) are currently involved in a primary study potentially eligible for inclusion in this review. However, no authors have any vested interest in the outcomes of this review, nor any incentive to represent findings in a biased manner.

## SOURCES OF SUPPORT

**Internal sources**

- VIVE—The Danish Center for Social Science Research, Denmark

**External sources**

- No sources of support provided

## Supporting information

Supporting information.
