# Supplementary material for: PROTOCOL: School‐based language, math, and reading interventions for executive functions in children and adolescents: A systematic review
Source: Campbell Syst Rev. 2022 Jul 12;18(3):e1262. doi: 10.1002/cl2.1262 (PMC9275554; doi:10.1002/cl2.1262)
Supplement: Supplementary file 1 — Supporting information. [file CL2-18-e1262-s001.docx]

# Appendices

## 1 Screening guide

This appendix contains the guidance questions for the screening tool. Please note that this guidance is subject to adjustments during the screening phase. The screening process is double blinded in both title/abstract and in full text screening. If screening disagreements cannot be resolved by the two screeners, one of the review authors will act as a third and final screener.

##### First level screening on title and abstract

*Table A1: Screening tool for title and abstract screening*

| Include/Relevant for full text screening | Answer “yes” or “lack information” to all guidance questions 1-4 |
| --- | --- |
| Exclude/Not relevant for full text screening | Answer “no” to at least one of the guidance questions 1-4 |
| Need to discuss with project team | Use this code if the abstract presents information that you are uncertain of how to be judged. All abstracts in this category will be discussed and resolved at the following screener-status meeting. Please write uncertainties/questions regarding the reference in the information box in EPPI. |
| Note: A study might be eligible for exclusion in several domains. | |

**Question 1: Is the abstract written in Danish, English, German, Norwegian or Swedish?**

**Yes:** Include

**Lack information:** Include

**No:** Stop screening and exclude

*Guidance: Only studies providing abstracts in languages that at least two of the research members can understand are eligible for inclusion. In the current review, these languages are English, Danish, German, Norwegian, and Swedish.*

**Question 2:** **Are the participants in the study children in pre, primary or secondary school age (approximately 3-18 years)?**

**Yes:** Include

**Lack information:** Include

**No:** Stop screening and exclude

*Guidance: The population eligible for this review are students attending either pre, primary or secondary school, which in most countries will be equivalent to 3-4 years until 18 years of age.*

**Question 3:** **Does the study examine either a math, reading or language intervention?**

**Yes:** Include

**Lack information:** Include

**No:** Stop screening and exclude

*Guidance: This review is interested in reading, math or language skills interventions with the purpose of increasing skills in these academic areas.*

**Question 4: Does the study mention some kind of executive function?**

**Yes:** Include

**Lack information:** Include

**No:** Stop screening and exclude

*Guidance: Executive functions is a broad term. In this review we are interested in a number of different types of executive functions. Other terms describing executive functions are: inhibition, attention, working memory, verbal memory, impulse control, emotional control, flexible thinking, self-monitoring, task initiation, problem solving, concept formation, goal orientation, planning, prioritising, organising, self regulation, attention, inhibition.*

**Question 5: Does the study report quantitative measures?**

**Yes:** Include

**Lack information:** Include

**No:** Stop screening and exclude

*Guidance: This review does not include qualitative research. If studies explicitly state that they only use qualitative methods, then exclude. If the study is a review or meta-analysis within our research field, then include.*

##### Second level screening on full text

*Table A3: Full text screening tool*

| Include in review | Answer “yes” or “uncertain” to all guidance questions |
| --- | --- |
| Exclude from review: Wrong language | Answer “no” to question 6 (or to question 1) |
| Exclude from review: Wrong setting | Answer “no” to question 7 (or to question 2) |
| Exclude from review: Wrong intervention | Answer “no” to question 8 (or to question 3) |
| Exclude from review: Wrong type of tests | Answer “no” to question 9 (or to question 4) |
| Exclude from review: Wrong study design | Answer “no” to question 10 (or to question 5) |
| Note 1: A study might be eligible for exclusion in several domains. Choose the first found reason for exclusion or the most dominant reason for exclusion.  Note 2: Studies included with an “uncertain”-judgement should be sent to one of the review authors for final assessment of eligibility. | |

**Question 6: Is the full text written in Danish, English, German, Norwegian or Swedish?**

**Yes:** Include

**Uncertain:** Include, and send to one of the review authors

**No:** Stop screening and exclude

*Guidance: Studies might provide abstracts in English, while the full text is written in another language. Only studies providing full texts in languages that at least two of the research members understand are eligible for inclusion. In this review, eligible languages are English, Danish, Swedish, and Norwegian.*

**Question 7: Does the intervention take place in a school setting?**

**Yes:** Include

**Uncertain:** Include, and send to one of the review authors

**No:** Stop screening and exclude

*Guidance: Interventions must be implemented within a pre, primary or secondary school setting. School settings does not include summer schools and after-school programs. We will make no restrictions on types of schools in which the interventions are performed, thus both regular schools and special school settings are eligible for inclusion.*

*Terminology of primary and secondary school*

*Within the English speaking world, there are three widely used systems to describe the grade and corresponding age of the child in the educational system: The American model, the English model, and the 'equivalent ages' of the student. This terminology extends into the research literature. See table A1 for a convenient comparison between the three models.*

*Table A2: Comparison between school models (grades, forms and ages)*

| Equivalent ages | US grades | US nicknames | England grades | England forms |
| --- | --- | --- | --- | --- |
| 3-4 years old | Preschool | - | Preschool | - |
| 4-5 years old | Preschool  Pre-K | - | Preschool  R | Reception |
| 5-6 years old | K | - | 1 | Infants |
| 6-7 years old | 1 | - | 2 | Top infants |
| 7-8 years old | 2 | - | 3 | Junior 1 |
| 8-9 years old | 3 | - | 4 | Junior 2 |
| 9-10 years old | 4 | - | 5 | Junior 3 |
| 10-11 years old | 5 | - | 6 | Junior 4 |
| 11-12 years old | 6 | - | 7 | First |
| 12-13 years old | 7 | - | 8 | Second |
| 13-14 years old | 8 | - | 9 | Third |
| 14-15 years old | 9 | Freshman | 10 | Fourth |
| 15-16 years old | 10 | Sophomore | 11 | Fifth |
| 16-17 years old | 11 | Junior | 12 | Lower sixth |
| 17-18 years old | 12 | Senior | 13 | Upper sixth |

**Question 8: Is the intervention aimed at specific content domains in order to improve either numeracy, literacy or language skills?**

**Yes:** Include

**Uncertain:** Include, and send to one of the review authors

**No:** Stop screening and exclude

*Guidance: For an intervention to be eligible, the intervention must target a specific skill or skills within one of those domains. Such skills can be (pre-)algebra, geometry, probability, number sense, decoding, phonological awareness, vocabulary, comprehension etc.*

**Question 9: Are some of the outcome measures focused on executive functions?**

**Yes:** Include

**Uncertain:** Include, and send to one of the review authors

**No:** Stop screening and exclude

*Guidance: The intervention must report results related to executive functions. Executive functions need not be the sole purpose of the study. We will only use standardised measures of executive functions.*

**Question 10: Is the study an RCT or QES with more than one unit in treatment/control conditions?**

**Yes:** Include

**Uncertain:** Include, and send to one of the review authors

**No:** Stop screening and exclude

*Guidance: We are interested in randomised controlled trials (RCT) or quasi-experimental studies (QES), which use a control/comparison design to examine effects. Such studies can have many labels, so it is important to read the study design section in order to make a qualified assessment. The most common sub-categories of randomised controlled trials and quasi-experimental studies can be found in table A4 (please note that this is not a complete list).*

*Table A4: Most common study designs*

| **Randomised controlled trials (RCTs)** Participants are allocated at random to control and treatment groups. | **Quasi-experimental studies (QES)** Participants are not allocated at random to control and treatment groups. |
| --- | --- |
| Individual randomised assignment:  Individuals are assigned to treatment and control conditions at random (e.g., by coin toss or random number generator).  Cluster randomised assignment:  Groups are allocated to treatment and control conditions, e.g. classes or schools.  Stratified/blocked random assignment:  The sample is divided into stratas/blocks (e.g., grade levels), and then randomly assigned to treatment and control conditions within these stratas/blocks. | Pseudo-randomisation:  Non-random sequences used for assignment to treatment/control conditions (e.g., birth date, case number, alphabetic order etc.).  Matching:  Individuals are matched in pairs/groups on observed characteristics (e.g., pre-test scores or age).  Other possible designs:  Cohort studies, Regression-discontinuity design, instrumental variable design, case-control studies. |
| *Note: This is not an exhaustive list of study designs. if in doubt, please ask one of the review authors for further clarification.* | |

## 2 Codebook

| **Theme** | **Data to be extracted** |
| --- | --- |
| *Publication characteristics* | Author names  Publication year  Publishing status  Publication type |
| *Study Characteristics* | Study location  Study design  Number of sites in study  Type of school setting  Implementation quality |
| *Participant Characteristics* | Gender distribution  Age  Grade level  Ethnicity  Socio Economic Status  Diagnoses |
| *Intervention Characteristics* | Treatment name  Brief description of treatment modality  Skills targeted  Duration  Intensity  Frequency  Type of control |
| *Outcome Characteristics* | Primary outcome:  Test name  Type of measure  Test domain  Sample sizes  Type of test statistic  Test statistic  Secondary outcome:  Test name  Test domain  Sample sizes  Type of test statistic  Test statistic |
